# Synthesis and Characterization of New Biodegradable Injectable Thermosensitive Smart Hydrogels for 5-Fluorouracil Delivery

**DOI:** 10.3390/ijms22158330

**Published:** 2021-08-03

**Authors:** Adam Kasiński, Monika Zielińska-Pisklak, Sebastian Kowalczyk, Andrzej Plichta, Anna Zgadzaj, Ewa Oledzka, Marcin Sobczak

**Affiliations:** 1 Department of Biomaterials Chemistry, Chair of Analytical Chemistry and Biomaterials, Faculty of Pharmacy, Medical University of Warsaw, 1 Banacha St., 02-097 Warsaw, Poland; adam.kasinski@wum.edu.pl (A.K.); mpisklak@wum.edu.pl (M.Z.-P.); ewa.oledzka@wum.edu.pl (E.O.); 2 Faculty of Chemistry, Warsaw University of Technology, 3 Noakowskiego Str., 00-664 Warsaw, Poland; skowalczyk@ch.pw.edu.pl (S.K.); aplichta@ch.pw.edu.pl (A.P.); 3 Department of Environmental Health Sciences, Faculty of Pharmacy, Medical University of Warsaw, 1 Banacha St., 02-097 Warsaw, Poland; azgadzaj@wum.edu.pl

**Keywords:** biomedical hydrogels, ε-caprolactone and poly(ethylene glycol) copolymers, antitumor drug delivery systems, local drug delivery systems, smart hydrogels

## Abstract

In this paper, injectable, thermosensitive smart hydrogel local drug delivery systems (LDDSs) releasing the model antitumour drug 5-fluorouracil (5-FU) were developed. The systems were based on biodegradable triblock copolymers synthesized via ring opening polymerization (ROP) of ε-caprolactone (CL) in the presence of poly(ethylene glycol) (PEG) and zirconium(IV) acetylacetonate (Zr(acac)_4_), as co-initiator and catalyst, respectively. The structure, molecular weight (M_n_) and molecular weight distribution (*Đ*) of the synthesized materials was studied in detail using nuclear magnetic resonance (NMR) and gel permeation chromatography (GPC) techniques; the optimal synthesis conditions were determined. The structure corresponded well to the theoretical assumptions. The produced hydrogels demonstrated a sharp sol–gel transition at temperature close to physiological value, forming a stable gel with good mechanical properties at 37 °C. The kinetics and mechanism of in vitro 5-FU release were characterized by zero order, first order, Higuchi and Korsmeyer–Peppas mathematical models. The obtained results indicate good release control; the kinetics were generally defined as first order according to the predominant diffusion mechanism; and the total drug release time was approximately 12 h. The copolymers were considered to be biodegradable and non-toxic; the resulting hydrogels appear to be promising as short-term LDDSs, potentially useful in antitumor therapy.

## 1. Introduction

Tumor diseases are one of the most difficult challenges in modern medicine. Despite various available treatment methods and many years of research in this field, the effectiveness of known therapies remains insufficient. Tumors are the world’s second leading cause of death, according to World Health Organization data [1]. Chemotherapy, one of the most widely used antitumor treatments, is associated with high and non-specific cytotoxicity of antitumor drugs when administered intravenously. Cytostatic doses must be limited due to severe systemic side effects, and they frequently do not provide effective treatment [2]. One strategy for increasing therapy effectiveness is to limit the distribution of cytostatics to the target tissue, which reduces systemic toxicity while also ensuring a sufficient drug concentration in the tumor. This strategy can be carried out by employing novel polymeric local drug delivery systems (LDDSs) [3,4], such as micro- and nanoparticles [5,6], micelles [7,8], or hydrogels [7,8]. Hydrogels are defined as three-dimensional, crosslinked structures able to absorb large amounts of water or body fluids while maintaining integrity [9,10]. In recent years, much attention has been paid to stimuli-responsive hydrogels, so-called smart hydrogels, as potential LDDSs. Temperature, pH, light, magnetic field, or biological factors such as enzymes or antibodies could cause these biomaterials’ properties to change. The drug release from these hydrogels can be remotely controlled or triggered by the factors mentioned above. Whereas, injectable hydrogels are in situ gel-forming matrices that are administered as a free-flowing sol into the target tissue and form a stable gel at physiological conditions due to crosslinking processes. The most significant benefits of these systems are: ease of implantation, protection of the implantation site against infection, and increased patient comfort and compliance. Furthermore, because of local implantation, drug distribution is generally limited to the target tissue; therapy effectiveness is enhanced while side effects are minimised [9,10,11,12,13].

One of the strategy for developing injectable hydrogels is to obtain thermosensitive hydrogels undergoing sol–gel transition at physiological temperature. In particular, at room temperature, the polymer aqueous solution forms a free-flowing sol. As the temperature rises to the lower critical solution temperature (LCST), the viscosity of the hydrogel rapidly increases, forming a stable gel. As the temperature rises to the upper critical solution temperature (UCST), the gel–sol transition occurs and the hydrogel starts to flow [10,14,15]. 

Polyesters such as poly(ε-caprolactone) (PCL), polylactide (PLA), and polyglycolide (PGA) have received a lot of attention in the field of thermosensitive hydrogels for biomedical applications. Their biocompatibility and biodegradability distinguish them as potential LDDSs; they are nontoxic and do not require surgical resection after use [16]. Furthermore, block copolymers composed of PCL and poly(ethylene glycol) (PEG) exhibit LCST value close to physiological temperature, making them potentially useful as injectable hydrogel drug delivery systems (DDSs); the LCST value can be adjusted by varying the copolymer number average molecular weight (M_n_), CL/PEG molar ratio, and block copolymer topology [17]. In addition, these polymers are approved by the FDA [18]. Because PCL/PEG triblock copolymers are composed of hydrophilic PEG blocks (B) and hydrophobic PCL blocks (A), they can be divided into BAB (PEG-*b*-PCL-*b*-PEG, PECE) and ABA (PCL-*b*-PEG-*b*-PCL, PCEC) types with slightly different properties [19]. The PCEC copolymer has a wider gel window, a longer in vivo persistence (about three weeks), and can be synthesized in a single step without the use of any coupling agents [3]. The PCEC sol–gel–sol transition mechanism can be explained by micelles formation and aggregation. Because of the amphiphilic nature of PCEC copolymer, the macromolecules self-assemble into micelles in aqueous media at ambient temperature. As the temperature rises, the diameter of the micelles rises, and the interactions between micelles become stronger; intermicellar bridges form as well. Furthermore, as the temperature approaches the LCST value, hydrophobic interactions between copolymer molecules are thermodynamically preferred over copolymer-water interactions, and the sol–gel transition occurs. When the temperature reaches the UCST value, the micelles disintegrate, the copolymer precipitates, and the hydrogel starts to flow [13,20].

Ring-opening polymerization (ROP) is the most widely used method for producing PCL/PEG block copolymers. Due to its high reactivity, the vast majority of papers describe the procedure using stannous octoate (Sn(Oct)_2_) as a catalyst and PEG-diol as a co-initiator. However, there are some concerns about tin-based catalysts’ biosafety; they can be neurotoxic and cause DNA damage [21]. Thus, in the biomedical field, a tin-free catalyst effective in ROP of cyclic esters is highly desirable [19,22]. Zirconium(IV) acetylacetonate (Zr(acac)_4_) appears to be a promising alternative to Sn(Oct)_2_ as a ROP catalyst. It has a low toxicity and excellent catalytic activity in the polymerization of ε-caprolactone (CL), lactide (LA), and glycolide (GA). Furthermore, Zr(acac)_4_ has been approved by the FDA as a drug and cosmetics additive, and it is less toxic than tin-based catalysts [21,22,23].

To date, various PCEC and PECE hydrogel formulations have been utilized as injectable DDSs [12,24,25,26,27,28,29,30]. These poly(ether-ester)s show excellent biodegradability and biocompatibility. Moreover, their aqueous solutions exhibit rapid sol–gel transition upon heating at the temperature slightly below physiological and form stable gel with good mechanical properties [13,19]. Khodaverdi et al. [12] obtained a PCEC injectable hydrogel depot as growth hormone (hGH) delivery system. The in vitro drug release assay revealed the hGH was released in prolonged and controlled manner up to three weeks. The release kinetics was denoted as first order with reduced burst release observed at the initial stage of the experiment. Analysing the results of Korsmeyer–Peppas and Higuchi mathematical modelling, the mechanism of the release was denoted as non-Fickian transport, with a predominance of diffusional mechanisms. Patel et al. [24] developed a blended injectable hydrogel DDS comprising PCEC and PECE copolymers. The copolymers were successfully synthesized via ROP using Sn(Oct)_2_ as catalyst; the synthesis of PECE required the coupling reaction with hexamethylene diisocyanate at the second step of the reaction. In vitro drug release profile of diclofenac sodium indicated the prolonged and controlled manner of the release. The mathematical modelling revealed the diffusional mechanism of the release (*n* coefficient for Korsmeyer–Peppas model was 0.40). The release profile followed first order kinetics up to 14 days. For comparison, the release profile of free drug (in the absence of hydrogel) was only about 3 h; the release was prolonged over 100 times.

The evidence concerning PCEC injectable hydrogels as 5-fluorouracil (5-FU) DDSs is strongly limited [29,31]; the kinetics of the release and the release mechanism were not defined. Moreover, the copolymers were synthesized using tin-based catalysts.

As a result of the foregoing, injectable smart hydrogels with potential applications as LDDSs were produced in this study by using biodegradable PCEC triblock copolymers as the gel-forming agent. As an alternative to commonly used tin-based catalysts, the copolymers were synthesized using ROP of CL in the presence of PEG and Zr(acac)_4_; Zr(acac)_4_ has a significantly improved biosafety profile. The structure of the obtained PCEC copolymers was thoroughly examined, and the ROP reaction parameters were optimized; the gelling behavior and rheological properties of PCEC hydrogels were also evaluated. Furthermore, the in vitro drug release profiles of 5-FU, a model hydrophilic antitumor drug, were determined along with kinetics and drug release mechanism characterization using a mathematical model. 

Despite extensive research into injectable smart hydrogel LDDSs, such a system has yet to be commercialised and used in clinical practise. As a result, the innovative smart hydrogel LDDSs presented in this paper appear to be promising. We hope that the resulting hydrogel carriers can be used in practise as controlled 5-FU delivery systems.

## 2. Results and Discussion

### 2.1. Synthesis and Structural Analysis of Copolymers

The primary goal of the study was to create a PCEC-based injectable and thermosensitive smart hydrogels and assess their suitability as a 5-FU delivery system. Furthermore, the suitability of Zr(acac)_4_ as a catalyst for ROP of CL in the presence of PEG has been investigated. 

The optimal conditions for ROP (reaction time and CL/catalyst molar ratio) were specified in the first step of our research, for which the agreement between experimental and theoretically calculated M_n_ of copolymers was the closest to 100% and the product was obtained with high yield and high CL conversion. Simultaneously, the optimal M_n_ of PEG and CL/PEG ratio [m/m] were specified, providing the gel-forming properties of the obtained copolymer with LCST close to 37 °C.

**Scheme 1:**
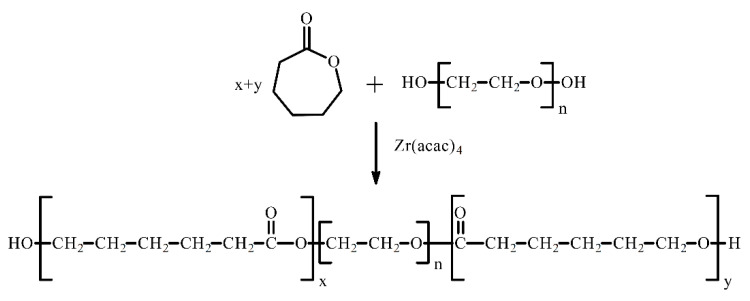
The ROP of CL in the presence of PEG and Zr(acac)_4_.

A series of PCEC triblock copolymers were synthesized via ROP process (Scheme 1). The copolymers were varied in CL/PEG (m/m) ratios and PEG molecular weight. Table 1 contains the data gathered. All of the samples were synthesized from 0.5 g of PEG characterized by various M_n_. The molar ratio of CL to catalyst was 100:1. The copolymerization process was carried out at a temperature of 130 °C.

**Figure 1:**
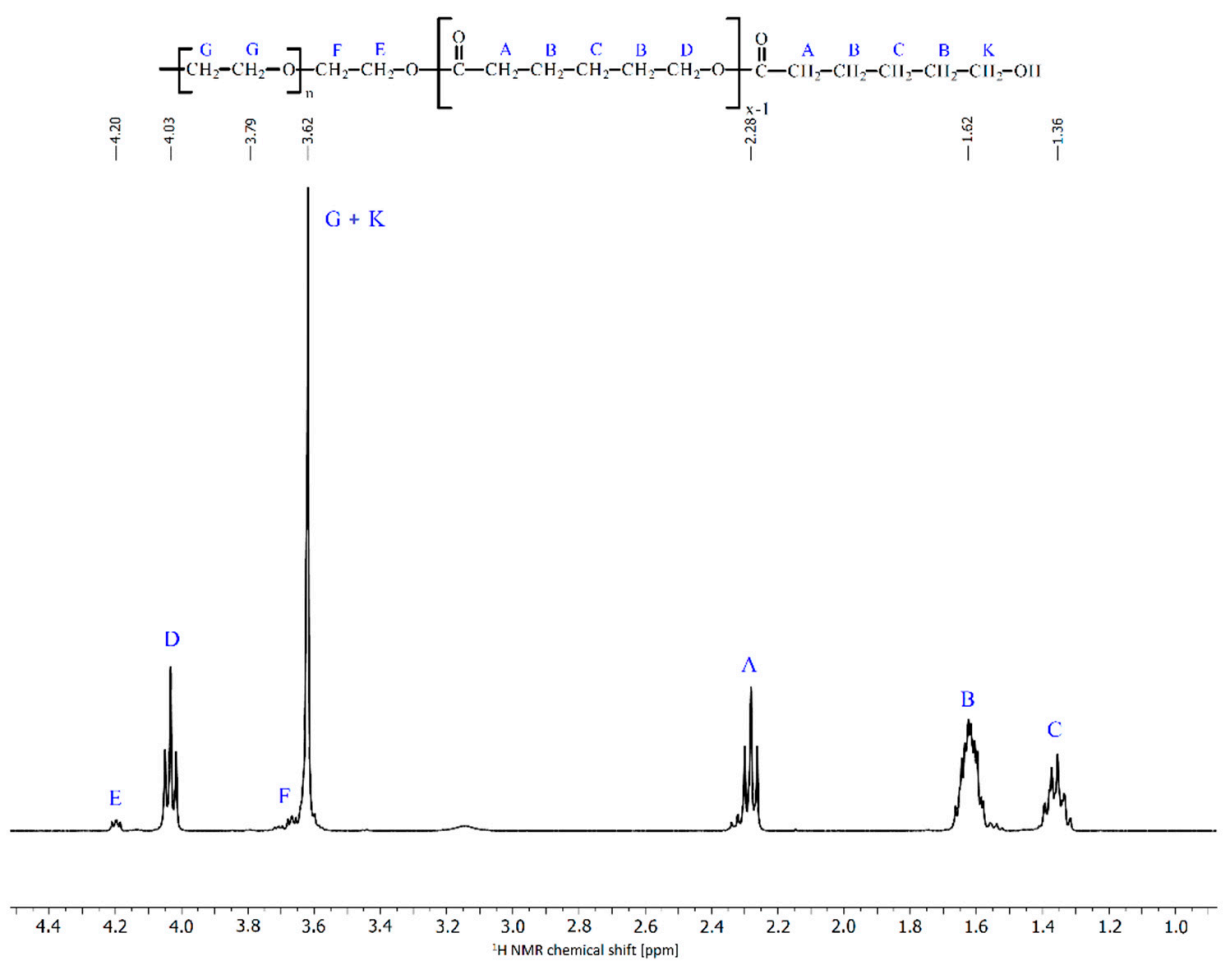
^1^H NMR spectrum of PCEC triblock copolymer.

**Figure 2:**
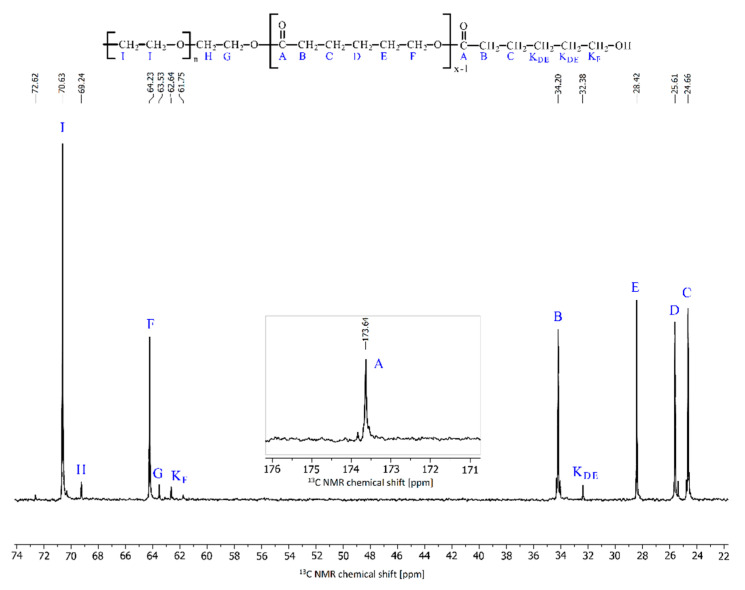
^13^C NMR spectrum of PCEC triblock copolymer.

The structure of the PCEC copolymers was confirmed using ^1^H and ^13^C NMR. The copolymer’s characteristic signals were clearly visible on the spectra and were assigned to specific atoms (see experimental section). Figure 1 and Figure 2 show examples of spectra. The spectroscopic data was consistent with the literature data [32].

The molecular weight and PCL block size were found to be in good agreement with theoretical values, though the percentage of agreement varied depending on the M_n_ of the PEG and the theoretical M_n_ of the copolymers calculated from the feed ratio (Table 1). The agreement between measured and theoretical values of M_n_ of copolymers increased significantly as M_n_ of PEG increased from 200 g/mol to 1000 g/mol. When the M_n_ content of copolymers was increased to 2000 g/mol, similar results were obtained. Above this point, the percent of agreement appears to be independent of M_n_ of PEG, reaching a plateau around 125%. Consideration of the agreement of the PCL block size with theoretical values reveals converging conclusions. When the copolymer M_n_ is low, a slight difference between theoretical and experimental data results in a relatively high percent agreement value. This effect became less pronounced as M_n_ values increased. When the relatively high molecular weights of the synthesized copolymers are considered, it can be concluded that the M_n_ values (calculated from ^1^H NMR spectra) were consistent with the theoretical values calculated from the CL and PEG feed ratios, and the control over the polymerization process was adequate.

**Figure 3:**
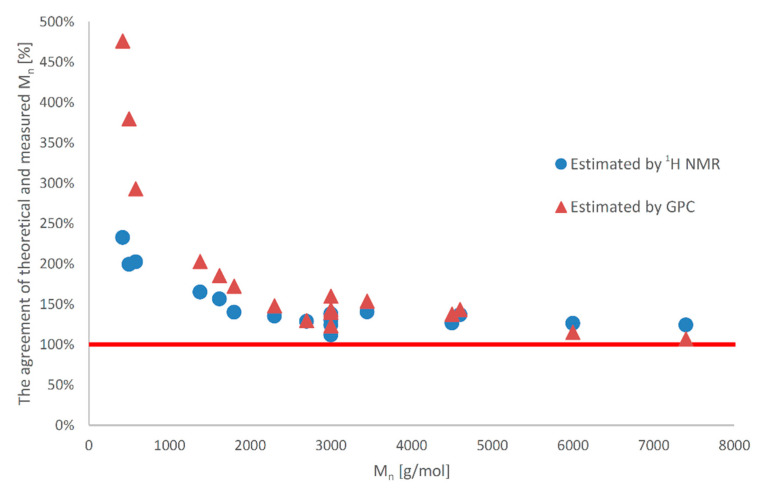
The relationship between the percent agreement of the estimated M_n_ and the theoretical M_n_.

**Figure 4:**
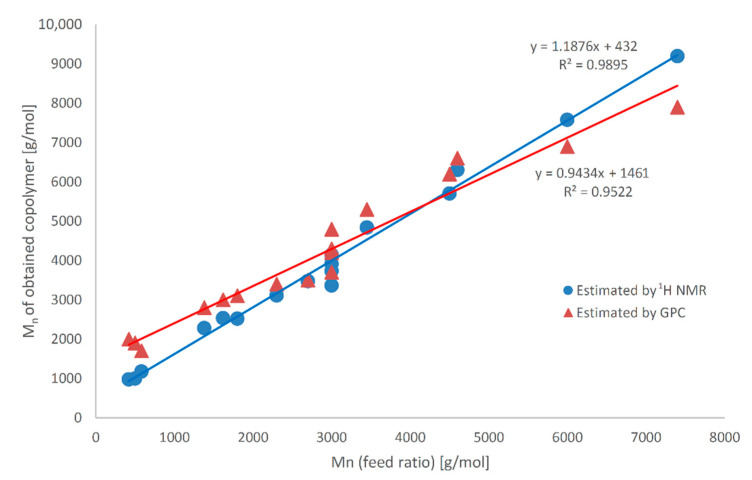
Estimated M_n_ of PCEC versus theoretical M_n_ values calculated from feed ratio.

Figure 3 describes the relationship between the percent agreement of the estimated M_n_ and the theoretically calculated M_n_. The dependence of M_n_ of the obtained copolymer was determined to be directly proportional to M_n_ calculated from the feed ratio; the relationship appears to be linear (Figure 4), indicating that the polymerization was carried out in a well-controlled manner. Furthermore, a comparison of samples synthesized under identical conditions, namely PCEC-7 and PCEC-9, revealed that the process was repeatable.

The M_n_ and dispersity *(Đ*) of the obtained PCEC triblock copolymers were determined by gel permeation chromatography (GPC) (Table 1). The M_n_ values were in satisfactory agreement with theoretical and estimated by ^1^H NMR values, especially for copolymers with a M_n_ above 2000 g/mol. Moreover, the synthesized copolymers were characterized by relatively narrow *Đ* index (ranged from 1.45 to 1.60). 

A series of PCEC copolymers were synthesized to determine the optimal CL/catalyst ratio. According to results mentioned above and preliminary tests of hydrogels gel-forming properties (Table 1 and Figure 3), the M_n_ of PEG was set at 1000 g/mol, and the CL/PEG ratio was 1.3, 1.7, or 2.0 [m/m]; the reaction time and temperature were 6 h and 130 °C, respectively. Under these conditions, the obtained copolymers exhibit gel-forming properties with LCST close to 37 °C. Furthermore, the percentage agreement between theoretical and experimental M_n_ calculations was close to 100%.

The CL/catalyst molar ratio of the prepared samples ranged from 100:1 to 1250:1. Table 2 displays the results of the synthesis.

**Figure 5:**
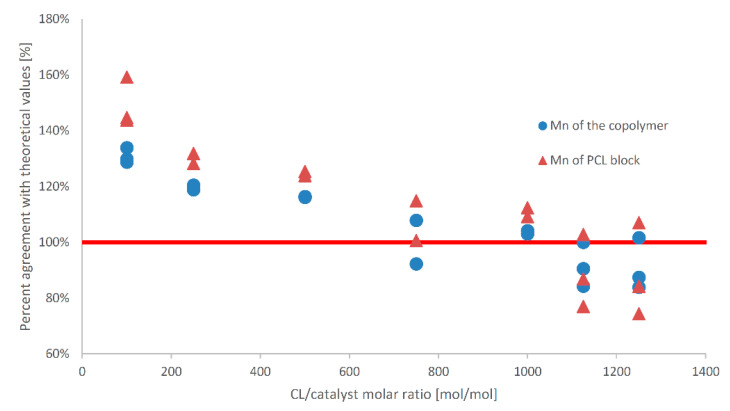
The percent agreement of M_n_ of the copolymers and the PCL block versus CL/catalyst molar ratio.

When the agreement of M_n_ of copolymers with the theoretical values calculated from feed ratio was compared, a decrease in percent agreement was observed with a decrease in catalyst amount from approximately 130% for 100:1 [mol/mol] to approximately 90% for 1250:1 [mol/mol]. The M_n_ agreement increased to the limit value (at 1000:1) as the catalyst amount was decreased; further reduction of the catalyst amount worsened the agreement, and the obtained M_n_ values were below theoretical assumptions (Figure 5). Furthermore, no significant relationship was perceived between the aforementioned agreement and the CL/PEG ratio. Based on these findings, the optimal CL/Zr(acac)_4_ molar ratio was determined to be 1000:1; the percentage agreement of M_n_ of copolymers between theoretical and experimental calculations appears to be the closest to 100% (Table 2 and Figure 5).

To evaluate the polymerization reaction kinetics, a series of PCEC copolymers were synthesized in variable time periods using PEG 1000 at 130 °C; the monomer/catalyst molar ratio was 1000:1 and the CL/PEG ratio was 2:1 [m/m] (Table 3).

**Figure 6:**
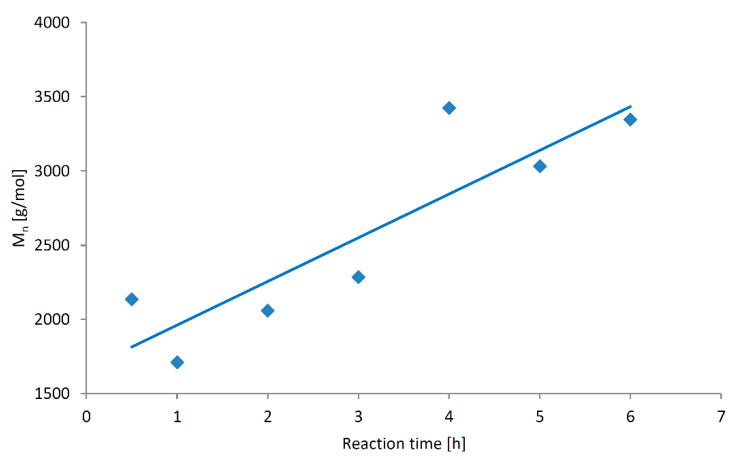
The relationship between the M_n_ of PCEC and the reaction time.

It was discovered that the conversion, products yield and M_n_ as well *Đ* increased concurrently with the reaction time; the relationship between M_n_ and the reaction time is presented in Figure 6. Zr(acac)_4_ appears to be an effective catalyst for ROP of CL in the presence of PEG as a co-initiator; the conversion value was high even though the reaction time was very short (89% of conversion after 0.5 h). The results of the reaction yield interpretation are fairly convergent, but the observed products yield values are significantly lower when compared to conversion. It is assumed that the M_n_ of the PCL blocks in triblock copolymer is relatively low at the initial stage of the reaction when compared to M_n_ of PEG; the hydrophilicity of the macromolecule is high, and thus a significant amount of the product is probably washed out at the purification stage.

The optimal synthesis conditions were determined by examining the structure of the obtained PCEC copolymers as well as the gelation behaviour of hydrogels. Under these conditions, the copolymers exhibit gel-forming properties at the desired temperature, and the percentage agreement between M_n_ of PCEC (as well as M_n_ of PCL blocks) were close to 100%. Following that, the PCEC triblock copolymers were synthesized on a larger scale, using 4.0 g of PEG 1000 and 6.8 g and 8.0 g of CL for PCEC-A1.7 and PCEC-A2.0, respectively. The reactions were carried out in 130 °C for 6 h; with a molar ratio of 1000:1 of CL/Zr(acac)_4_. Table 1 shows again the structural parameters of the obtained copolymers.

### 2.2. Sol–Gel–Sol Phase Transition and Rheological Properties

The sol–gel–sol transition of thermosensitive PCEC hydrogels was investigated using the tube inverting method. According to the data in Table 1, when the M_n_ of the PEG block was below 1000 g/mol, the PCEC copolymers were insoluble in an aqueous environment and did not form a homogeneous sol; the M_n_ of the copolymers was too low to form stable micelles. Furthermore, the hydrophobic/hydrophilic moieties ratio was relatively high, limiting the copolymer’s water solubility. For hydrogels obtained from PEG 1500 and PEG 2000, the gel–sol transition was not observed; the mixtures remained undissolved or formed a transparent sol up to the temperature of precipitation.

**Figure 7:**
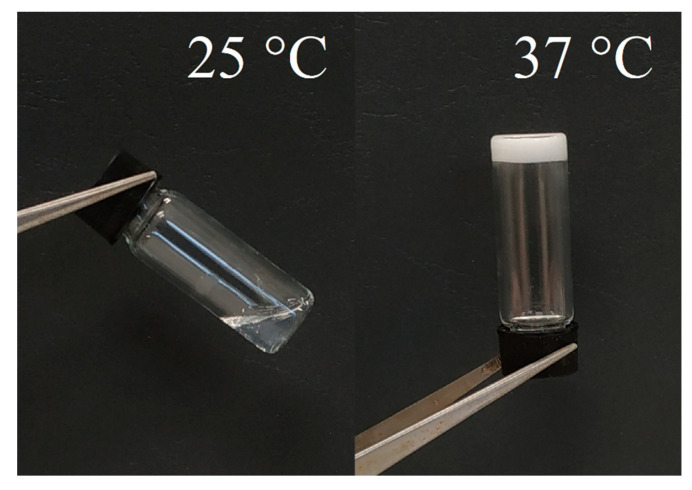
The example of thermosensitive hydrogel phase transition (PCEC-A2.0, concentration 20 wt%).

The temperature-dependent gelation behavior of hydrogels was observed when PEG 1000 was used as the polymerization co-initiator, and the LCST value was close to physiological temperature. Specifically, when the temperature reached LCST, the sol became opaque and formed a stable gel with good mechanical properties (Figure 7). After analysing the PCEC structure, it was determined that the LCST is primarily determined by the PCL/PEG ratio and the M_n_ of the copolymer. In brief, increasing the hydrophobic/hydrophilic balance decreases the gelation temperature and limits the system’s water solubility. When the hydrophobic block size increases, the hydrogel is hydrated less effectively, allowing the sol–gel transition to occur more easily; the LCST decreases. On the other hand, it was determined that increasing the copolymer M_n_ increases the LCST. The PCEC micelles became more stable and exhibited the sol–gel transition at higher temperatures, or did not perform the transition at all. It is suspected that for higher M_n_ values there have fewer PCEC macromolecules in a solution of a given concentration than for lower M_n_ copolymers. 

In the described experiment, the M_n_ of PEG remained constant, thus an increase in PCL/PEG ratio was correlated with an increase in the copolymer M_n_. The effects of these two parameters are opposite to each other, therefore, it is difficult to define the LCST’s dependence on these parameters unanimously. However, based on the experimental data, it is possible to conclude that the optimal CL/PEG ratio is 1.7 or 2.0 [m/m], and these ratios were chosen for further investigation.

**Figure 8:**
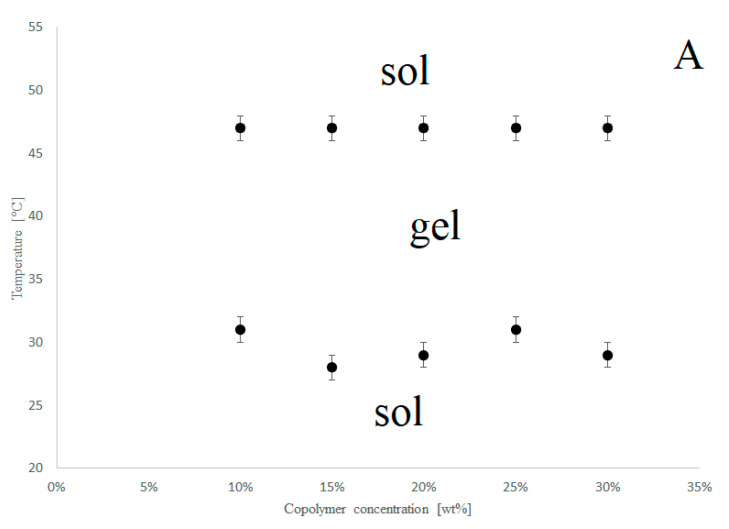

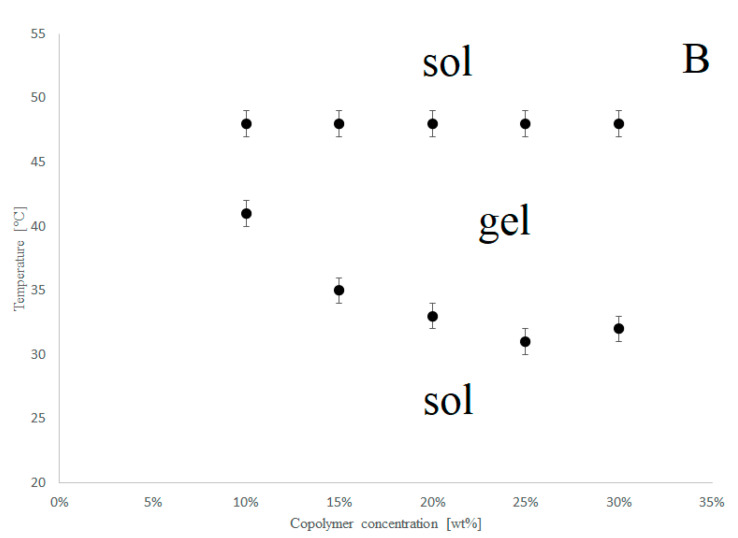
Phase transition diagrams of PCEC-A1.7 (**A**) and PCEC-A2.0 (**B**).

The relationship between the copolymer concentration and the LCST and UCST was evaluated for PCEC-A1.7 and PCEC-A2.0; the concentration range was 5–30 wt%. The phase transition diagrams were presented in Figure 8.

In general, it was discovered that as concentration increases, the gel window expands for Sample A; for Sample B the effect was not clearly observed (Figure 3). At higher concentrations, there are more PCEC macromolecules per volume unit, and the intermolecular interactions are stronger; micelles expand and can form a non-flowing gel at lower temperatures. Furthermore, at the same concentration, the LCST values of Hydrogel B are lower than those of Hydrogel A. This phenomenon could be explained by the fact that the M_n_ of the PCEC copolymer that forms Hydrogel B is slightly higher than that of Hydrogel A (3400 g/mol versus 3000 g/mol, respectively). It is well known that increasing the M_n_ of PCEC causes the LCST of the hydrogel to increase. The same phenomenon is seen with UCST, but it is less noticeable [17]. However, no significant relationships were found between copolymer concentration and UCST values. The gel–sol transition of PCEC hydrogels is thought to be caused by micelle disintegration, which leads to copolymer precipitation. Because the temperature of precipitation appears to be independent of polymer concentration, the relationship between UCST and copolymer concentration may be insignificant. 

**Figure 9:**
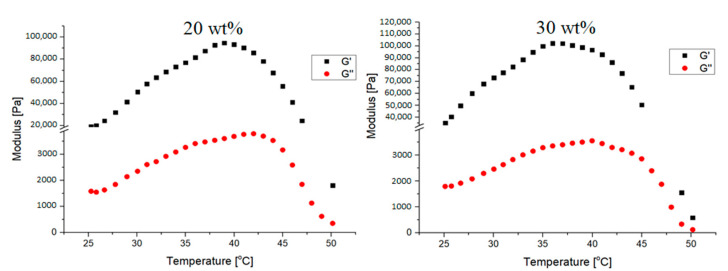
The G′ and G” moduli of PCEC-A2.0 aqueous solutions as a function of temperature.

Rheological analysis was used to examine the gelation properties of PCEC-based hydrogels. The values of storage modulus (G′) and loss modulus (G″) for PCEC-A2.0 aqueous solutions at two predetermined concentrations, namely 20 wt% and 30 wt%, were determined depending on temperature. It was clearly observed for each concentration that the values of the G′ increased abruptly and reached a maximum at 39 °C and 36 °C for 20 wt% and 30 wt% solutions, respectively. The maximum G′ values for a 20 wt% solution were 94,700 Pa and 102,200 Pa for a 30 wt% solution. When the temperature goes up above 42 °C, the moduli values drop significantly due to micelle disorganisation and gel–sol transition. Figure 9 shows the relationship between G′ and G” for each concentration as a function of temperature. The results showed that the hydrogels have good mechanical properties as potential injectable DDS; it is a free-flowing sol at room temperature and forms a stable gel at physiological temperature.

### 2.3. Cytotoxicity and Genotoxicity Assay

Cytotoxicity assay of the PCEC copolymers was performed using the neutral red uptake (NRU) test. It was observed that for the highest concentrations of the extracts tested (1 mg/mL), the cell survival rate was 96% and 98% for PCEC-A1.7 and PCEC-A2.0, when compared to the negative control, respectively (Table S1, Supplementary Materials). These results indicate that the tested hydrogels have no cytotoxic effect.

The umu-test with and without metabolic activation was used to assess the toxicity of PCEC copolymers. At the highest concentration tested (1 mg/mL), none of the samples tested inhibited the growth of *Salmonella typhimurium* cells (G > 0.5). Furthermore, the induction ratio (IR) for both tested samples was significantly lower than 1.5; genotoxicity was not observed for the hydrogels tested (Table S2, Supplementary Materials). 

### 2.4. Antitumor Drug Release Profiles

To estimate the drug release properties of developed injectable smart hydrogels and define their potential as antitumor DDSs, the drug release profiles were evaluated; 5-FU was the model hydrophilic drug with antitumor activity. 

**Figure 10:**
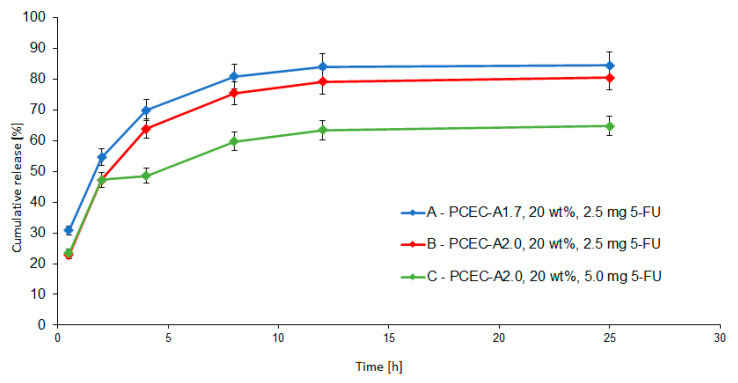
The 5-FU release profile from PCEC hydrogels: A (PCEC-A1.7, 25 wt%, 2.5 mg 5-FU); B (PCEC-A2.0, 20 wt%, 2.5 mg 5-FU); C (PCEC-A2.0, 20 wt%, 5.0 mg 5-FU).

The drug release profiles of two different polymeric matrices, PCEC-A1.7 (Sample A) and PCEC-A2.0 (Samples B and C), with different CL/PEG [m/m] ratios, were investigated. The 5-FU content was 2.5 mg/mL for A and B Hydrogels and 5.0 mg/mL for C hydrogel. The drug release profiles were presented as a relationship between the cumulative release and time (Figure 10).

The 5-FU-loaded hydrogels were in a sol state at room temperature; were transparent, free-flowing, and easy to inject with a 22 Gauge needle. Within 2 min of being placed at 37 °C, the solutions formed a stable, opaque gel.

After analysing the drug release profiles, it was determined that the release was complete after 25 h; the release rate decreased over time and reached a plateau after 12 h. Furthermore, no significant differences between Matrices A and B were observed; the copolymer structure of PCEC-A1.7 and PCEC-A2.0 had no effect on the release rate. While the Hydrogels B and C were compared, there was no significant relationship between the release profiles and the 5-FU concentration in the matrices.

To examine the drug release profiles in sufficient complexity, the obtained data were fitted to mathematical models, specifically the zero order, first order, Higuchi, and Korsmeyer–Peppas models. Table 4 summarises the findings. Because of the slight increase in cumulative release during the plateau phase, kinetic calculations were made using data from the first 12 h of the experiment.

Analyzing R^2^ values for zero order and first order kinetics, it was discovered that Hydrogels A and B had a 5-FU release profile similar to first order kinetics, which is typical for hydrophilic matrices releasing hydrophilic drugs. Furthermore, the drug release profiles were well-fitted to the Korsmeyer–Peppas model; R^2^ for A and B hydrogels was 0.999 and 0.998, respectively. The release exponent (*n*) was 0.397 (A) and 0.500 (B), indicating that the drug was released predominantly by diffusion (*n* ≈ 0.45). The predominance of diffusion mechanisms and first order kinetics is justified due to the matrix’s relatively long degradation time, significant hydrophilicity, and the hydrophilic nature of 5-FU itself. According to obtained results, it was concluded that the drug release rate is faster than the hydrogel degradation rate. As time passes, the path that the solvent penetrates into the pores of the insoluble matrix and the path through which 5-FU is eluted lengthens, and the concentration gradient decreases. This phenomenon causes the release rate to decrease in tandem with the drug release time, resulting in first order kinetics. The well-fitting of the release data from Samples A and B to the Higuchi model confirmed these findings (R^2^ was 0.915 and 0.920 for A and B, respectively). Similar results were obtained for Hydrogel C, with well-fitting for the Korsmeyer–Peppas model (R^2^ = 0.924, *n* = 0.331) and acceptable fitting for the Higuchi model (R^2^ = 0.878), indicating first order release kinetics with a predominant diffusion release mechanism.

**Figure 11:**
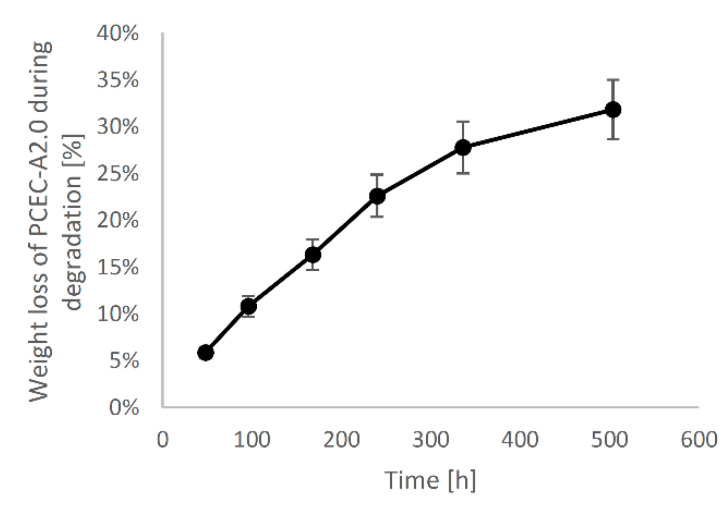
Hydrolytic degradation of PCEC-A2.0 hydrogel (20 wt%).

Simultaneously with the drug release experiment, hydrolytic degradation studies of the PCEC-A2.0 (20 wt%) hydrogel were carried out. The experiment was carried out in phosphate-buffered saline (PBS) (pH = 7.40 ± 0.05) for 504 h (21 days). Figure 11 shows the result.

After 48 h of incubation, the percent of degradation was around 6%, indicating that the drug release rate was significantly higher than the degradation studies. The degradation rate was approximately 32% at the end of the experiment (504 h). These findings are logical given that a mathematical model analysis of the 5-FU release results revealed a diffusive mechanism of the process. In our opinion, developed injectable hydrogels demonstrate effective properties as potential DDSs because of their biodegradability and long retention time in the body. However, it is important to note that the in vitro experiment is a simplified model of reality, and the results of the research cannot be easily translated into the LDDSs properties in the human body. In general, drug release rates in vivo are higher than in vitro experiments [33]. Despite the fact that the preliminary in vitro drug release properties of the obtained LDDSs are promising, additional research is undoubtedly required to confirm their utility for clinical use.

To the best of our knowledge, the evidence for similar hydrogels as 5-FU carriers derived from CL and PEG is extremely limited. As a result, it was difficult to provide adequate discussion with regard to a limited number of reference sources. Polymeric carriers of 5-FU, such as nanoparticles, nanocapsules, or nanoemulsions, have recently been developed and thoroughly studied [34,35,36,37,38]. Wang et al. [31] developed PECE triblock copolymer-based hydrogel DDSs capable of releasing 5-FU. The 5-FU release was relatively rapid; 26% of the drug was released in the first hour of the experiment and about 83% after 24 h; however, burst release was clearly observed. The hydrogel was biodegradable and injectable, though the gel window was narrower than that of the PCEC copolymer used in this study. Whereas, Gong et al. [29] developed hydrogel dual DDS that released paclitaxel (PTX) and 5-FU. The system was created by combining PTX-loaded nanomicelles with a 5-FU-containing PCEC aqueous solution. The in vitro 5-FU release assay revealed a high release rate (around 70% after 24 h) with significant burst release. However, the efficacy of the PTX-micelles-FU-hydrogel system was significantly increased when compared to combined treatment using free PTX plus 5-FU. The aforementioned literature findings certainly demonstrated the efficacy of formulations for anticancer 5-FU applications. However, the kinetics of 5-FU release from the aforementioned carriers have not been studied, and most formulations exhibit burst release at the initial stage. Furthermore, the polyester-based hydrogel DDSs were synthesized with the vast majority of cases utilising Sn(Oct)_2_ as a catalyst. Alternative solutions are highly desirable due to biosafety concerns about tin-based catalysts.

## 3. Materials and Methods

### 3.1. Materials

ε-Caprolactone (2-Oxepanone, CL, 97%, Aldrich, Poznan, Poland), zirconium(IV) acetylacetonate (tetrakis(2,4-pentanedionato)zirconium(IV), Zr(acac)_4_, 97%, Aldrich, Poznan, Poland), poly(ethylene glycol) 200 (PEG 200, M_n_ = 200 g/mol, pure, Fluka, Warsaw, Poland), poly(ethylene glycol) 600 (PEG 600, M_n_ = 600 g/mol, pure, Fluka, Warsaw, Poland), poly(ethylene glycol) 1000 (PEG 1000, M_n_ = 1000 g/mol, pure, Sigma-Aldrich, Poznan, Poland), poly(ethylene glycol) 1500 (PEG 1500, M_n_ = 1500 g/mol, pure, Sigma, Poznan, Poland), poly(ethylene glycol) 2000, (PEG 2000, M_n_ = 2000 g/mol, pure, TCI, Zwijndrecht, Belgium), dichloromethane (DCM, CH_2_Cl_2_, ≥99.8%, POCh, Gliwice, Poland), hydrochloric acid (HCl, ChemPur, Piekary Slaskie, Poland), Dulbecco’s Modified Eagle Medium (DMEM, Thermo Fisher Scientific, Warsaw, Poland), 5-fluorouracil (5-fluoropyrimidine-2,4-[1H,3H]-dione, 5-FU, 99%, Sigma-Aldrich, Poznan, Poland), phosphate-buffered saline (PBS, pH = 7.40 ± 0.05, ChemPur, Piekary Slaskie, Poland), phosphate-buffered saline for cell cultures (PBS, GIBCO, Dublin, Ireland), acetonitrile (ACN, HPLC grade min. 99.9%, POCH, Gliwice, Poland) and trifluoroacetic acid (TFA, 99%, Sigma-Aldrich, Poznan, Poland). Prior to use, PEGs compounds were heated in a vacuum for 2 h at 80 °C to remove residual water. Other reagents were used as received.

### 3.2. NMR Data

The ^1^H NMR spectrum of PCEC: 1.36 ppm (-O(O)C-CH_2_-CH_2_-C**H_2_-**CH_2_-CH_2_**-)**, 1.62 ppm (-O(O)C-CH_2_-C**H_2_**-CH_2_-C**H_2_**-CH_2_-), 2.28 ppm (-O(O)C-C**H_2_**-CH_2_-CH_2_-CH_2_-CH_2_-), 3.62 ppm (-O-C**H_2_**-C**H_2_**-) from PEG mers and (-O(O)C-CH_2_-CH_2_-CH_2_-CH_2_-C**H_2_**-OH end groups), 3.79 ppm (-O-C**H_2_**-CH_2_-O(O)C-CH_2_-CH_2_-CH_2_-CH_2_-CH_2_-), 4.03 ppm (-O(O)C-CH_2_-CH_2_-CH_2_-CH_2_-C**H_2_**-) and 4.20 ppm (-O-CH_2_-C**H_2_**-O(O)C-CH_2_-CH_2_-CH_2_-CH_2_-CH_2_-). 

The ^13^C NMR spectrum of PCEC: 24.66 ppm (-O(O)C-CH_2_-**C**H_2_-CH_2_-CH_2_-CH_2_-), 25.61 ppm (-O(O)C-CH_2_-CH_2_-**C**H_2_-CH_2_-CH_2_-), 28.42 ppm (-O(O)C-CH_2_-CH_2_-CH_2_-**C**H_2_-CH_2_-), 32.38 ppm (-O(O)C-CH_2_-CH_2_-**C**H_2_-**C**H_2_-CH_2_-OH) end groups, 34.20 ppm (-O(O)C-**C**H_2_-CH_2_-CH_2_-CH_2_-CH_2_-), 62.64 ppm (-O(O)C-CH_2_-CH_2_-CH_2_-CH_2_-**C**H_2_-OH) end groups, 63.53 ppm (-O-CH_2_-**C**H_2_-O(O)C-CH_2_-CH_2_-CH_2_-CH_2_-CH_2_-), 64.23 ppm (-O(O)C-CH_2_-CH_2_-CH_2_-CH_2_-**C**H_2_-), 69.24 ppm (-O-**C**H_2_-CH_2_-O(O)C-CH_2_-CH_2_-CH_2_-CH_2_-CH_2_-), 70.63 ppm (-O-**C**H_2_-**C**H_2_-) from PEG mers and 173.64 ppm (-O(O)**C**-CH_2_-CH_2_-CH_2_-CH_2_-CH_2_-).

### 3.3. Synthesis

The PCEC triblock copolymers were synthesized using the ROP reaction with Zr(acac)_4_ as the catalyst and PEG as the co-initiator, respectively. The reaction was carried out following the hot-melt method. In a brief, calculated amounts of Zr(acac)_4_ and PEG were placed in a glass ampoule and vacuum dried for several minutes to remove trace amounts of water. Following that, CL was added in an inert gas atmosphere using the Schlenk line, and the ampoule was tightly sealed. The reaction was carried out in an oil bath at 130 °C for the time specified (from 30 min to 24 h).

When the reaction was completed, the product was dissolved in DCM and purified with 5% HCl and distilled water to remove water-soluble impurities. The collected organic phase was then evaporated, yielding a waxy product that was vacuum dried for 24 h at 30 °C. Prior to use, the finished product was kept in a desiccator.

### 3.4. In Vitro 5-FU Release Studies

The 5-FU-loaded injectable hydrogels were prepared by dissolving the PCEC copolymer in 5-FU aqueous solution by heating-quenching method. Briefly, to form 20 wt% hydrogel 0.25 g of PCEC was mixed with 1.0 mL of 5-FU aqueous solution and heated up to 55–60 °C, which is above the melting point of PCL. In the next step, the mixture was quickly cooled in an ice bath, forming a transparent or cloudy free-flowing sol. The obtained hydrogels differed in terms of the copolymer structure (PCEC-A1.7 or PCEC-A2.0), polymer concentration (20 wt% or 30 wt%) and amount of 5-FU loaded (2.5 mg/mL or 5.0 mg/mL).

The membraneless method was used to investigate the in vitro drug release profiles [39]. In the first step, 1.0 mL of 5-FU-loaded free-flowing hydrogels were injected into vials using a 22 Gauge needle (20 mm × 40 mm). The matrices were then thermostated at 37 °C for 5 min to form opaque and semi-solid gels. The hydrogels were immersed in 5.0 mL of preheated PBS buffer (pH = 7.40 ± 0.05) and shaken at 60 rpm and 37 °C. After predetermined time intervals, the release medium was removed for further testing and completely replaced with 5.0 mL of preheated fresh PBS buffer. Prior to HPLC analysis, the collected samples of release medium were kept at −18 °C. The drug release studies were conducted for 168 h (7 days).

### 3.5. Hydrolytic Degradation

The PCEC hydrogel hydrolytic degradation assays were carried out simultaneously with drug release studies. Specifically, 1.0 mL of 20 wt% hydrogel in a sol state was injected into a 5.0 mL vial, allowed to gel for 5 minutes in 37 °C, and immersed in 5.0 mL of preheated PBS buffer. The matrices were incubated at 37 °C and gently shaken at 60 rpm. After predefined periods of time (i.e., 48 h, 96 h, 168 h, 240 h, 336 h and 504 h), the release medium was completely replaced, and the collected samples were dried in vacuum. The mass of the dry residues was used to calculate the weight loss of the examined copolymer, which corresponds to the degree of hydrolytic degradation. The weight of the degraded matrices was calculated using the following formula [40]:

(1) $$W=W_{d}-W_{o}-W_{p}$$

where *W* is the weight of the degraded matrices, *W_d_* is the weight of the dried vial containing the medium residues, *W_o_* is the weight of the original vial weight and *W_p_* is the weight of phosphate salts.

### 3.6. Measurements

#### 3.6.1. Structural Analysis

The structure of the obtained PCEC copolymers was elucidated by ^1^H and ^13^C NMR techniques. The spectra were recorded using Varian 300 MHz (Palo Alto, Santa Clara, CA, USA) and Agilent Technologies 400 MHz (Santa Clara, Santa Clara, CA, USA) spectrometer. The M_n_ of PCL and *PEG* block copolymers and their total M_n_ were estimated using the following equations [32]:

For PCL blocks:

(2) $$\frac{2\left(2\left(x-1\right)\right)}{I_{A}}=\frac{4}{I_{E}}$$

For *PEG* block:

(3) $$\frac{4\left(y-2\right)+4}{I_{PEG}}=\frac{4}{I_{E}}$$

For *M_n_*:

(4) $$M_{n}=2\left(114x\right)+44y$$

where *x* is a half of a number of *CL* mers in the copolymer, *y* is a number of EG units of PEG, *I*_A_, *I*_E_ and *I*_PEG_ corresponds to integral intensities of signals from methylene protons of *CL* mers (-O(O)C-CH_2_-CH_2_-CH_2_-CH_2_-C**H_2_**-O-) at 4.06 ppm, methylene protons of EG adjacent to CL mer (-CH_2_-C**H_2_**-O(O)C-) at 4.28 ppm and methylene protons of EG units of PEG (-O-C**H_2_**-C**H_2_**-) at 3.65 ppm, respectively (the numbers of 114 and 144 refer to M_n_ of CL and EG units, respectively).

The *CL* conversion was calculated from ^1^H NMR spectra of post-reaction mixture according to the following formula:

(5) $$conv\;CL=\frac{I_{D}}{I_{D}+I_{\alpha }}$$

where *Iα* and *I_D_* correspond to integral intensities of signals from methylene protons adjacent to α carbon atoms of *CL* monomer and *CL* units in PCEC copolymer, respectively. 

The M_n_ and *Đ* of the obtained PCEC copolymers were measured by GPC. The Malvern Viscotek GPCMax TDA 305 (Malvern Panalytical, Malvern, United Kingdom) chromatograph equipped with Jordi Gel DVB mixed bed column (Jordi Labs, Mansfield, MA, USA) was used; the mobile phase (DCM) flow rate was set as 1 mL/min and the temperature was set as 30 °C. The samples were dissolved in DCM and the system was calibrated with polystyrene standards.

#### 3.6.2. Sol–Gel–Sol Transition of the Hydrogels

The tube-inverting method was used to calculate the LCST and UCST values of the hydrogels [41]. A total of 1.0 mL of free-flowing PCEC sol with a predetermined concentration (5–30 wt%) was placed in a 13 mm inner diameter vial and immersed in a thermostated water bath. The temperature was gradually increased from 25 °C to 55 °C at a rate of 1 °C/min. After each 1 °C increase in temperature, the sample was allowed to equilibrate for 30 s before inverting the vial and visually assessing the hydrogel state as “sol” or “gel,” depending on whether flow was observed within 30 s.

Rheological properties of the hydrogels were determined using rotational rheometer ARES (TA Instruments, New Castle, Delaware, USA). The hydrogel sample was placed between two parallel plates with a 25 mm diameter and 0.3 mm gap. The dynamic analysis of G′ at a constant frequency (1 Hz) with variable deformation value (γ) was performed in the first step. For further investigation, the γ was adopted from the range where the relationship between G′ and γ was linear, namely 0.6%. Following that, the G′ and G” were measured as a function of temperature; the temperature range was 25–50 °C, the heating rate was 1 °C/min with 1 °C sampling [42].

#### 3.6.3. Cytotoxicity and Genotoxicity Assay

Cytotoxicity of the PCEC matrices was evaluated by the NRU test using BALB/c T3T clone A31 mice fibroblasts cell line (American Type Culture Collection), according to ISO 10993-5:2009 Annex A guideline [43]. The cells were seeded in 96-well plates (15,000/100 µL) and incubated for 24 h (5% CO_2_, 37 °C, > 90% humidity) in DMEM medium with 10% bovine serum and 100 IU/mL penicillin and 0.1 mg/mL streptomycin. After incubation, the cells monolayer was assessed under a microscope. The supernatant medium was replaced by the PCEC extracts prepared by incubating the material in a of 1 mg/mL DMEM medium with 5% bovine serum for 24 h at 37 °C, with stirring; the extracts were sterilized on a syringe filter. The cytotoxicity of each extract was determined in four two-fold dilutions, each in triplicate. The cells were incubated for 24 h. Subsequently, the medium was removed from the cells, the wells were washed with PBS and 100 µL of fresh medium with neutral red was added to each well. After 2 h the medium was removed, the wells were washed with PBS and ethanol and acetic acid aqueous solution was added to each well to release the dye from the cells. The plates were shaken for 15 min and afterwards the red color intensity was measured spectrophotometrically (λ = 540 nm). Polyethylene film and latex were used as reference materials (with no cytotoxicity and highly cytotoxic, respectively). Samples were considered cytotoxic if they reduced cell survival below 70% when compared to the untreated cells (a baseline cell viability).

Genotoxicity of the PCEC matrices was evaluated by the umu-test using *Salmonella typhimurium* TA3515/psk1002 (Deutsche Sammlung von Mikroorganismen Und Zellkulturen GmbH, Braunschweig, Germany) cell lines according to ISO 13829:2000 guideline [44]. The experiment was conducted with and without metabolic activation (hepatic fraction S9, Xenometrics). Further, 2-Aminoanthracene and 4-nitroquinoline N-oxide were used as positive controls. In response to various types of DNA damage, the umuC gene in bacterial cells, which is a part of the SOS system, was induced. The cell strains were genetically modified and the activity of the umuC gene was linked to β-galactosidase synthesis; the other DNA regions connected with this synthesis were deleted. In the presence of the enzyme, colorless substrate ortho-nitrophenyl-β-galactoside was converted to yellow product which can be quantified colorimetrically (at 420 nm). Moreover, the growth factor (G) was evaluated by optical density measurements; the cytotoxicity of tested samples was assessed. The genotoxic potential was characterized by an induction ratio (IR), the ratio of β-galactosidase activity between tested sample and the negative control. The sample was considered genotoxic when the IR value was ≥1.5. 

All tested polymers were incubated in PBS buffer (GIBCO) for 24 h at 37 °C with shaking. All of the extracts were sterilised by filtration prior to the assay. Each sample was examined in two three-concentration dilution series (three data points for each concentration). The PBS solvent control was prepared in the same manner.

#### 3.6.4. HPLC Analysis

The concentrations of 5-FU released were determined using HPLC, as previously described by our team [8]. In brief, the Hitachi Chromaster chromatograph was used, which included a pump type 5160, autosampler model 5260, thermostat 5310, and DAD-detector UVD 5430. Phenomenex C-18 4 mm 3 mm precolumn and Phenomenex Luna C-18 25 cm 4.6 mm (particle size 5 m) column were used in the system. The mobile phase was made up of Solvents A and B, H_2_O + 0.1% TFA and ACN + 0.1% TFA, respectively; the gradient is shown in Table S3 (Supplementary Materials). The flow rate was set to 1.0 mL/min, the injection volume was set to 20 L, and the column temperature was set to 35 °C.

#### 3.6.5. Mathematical Models

The kinetics and mechanism of 5-FU release were determined by fitting experimental data to theoretical mathematical models, zero-order and first-order, Higuchi and Korsmeyer–Peppas, using the following equations:

Zero-order:

(6) F=kt

First-order:

(7) $$\log F=\log F_{0}-\frac{kt}{2.303}$$

Higuchi model:

(8) $$F=k\sqrt{t}$$

Korsmeyer–Peppas model:

(9) $$\frac{M_{t}}{M_{\infty }}=kt^{n}\;(\frac{M_{t}}{M_{\infty }}<0.6)$$

where *F* is the amount of drug released, *F_0_* is the initial concentration of 5-FU, *t* is the release time increment, *k* is the model constant, *M_t_*/*M*_∞_ is the fraction of 5-FU released during time *t* and *n* is the exponent in the Korsmeyer–Peppas model [45,46].

## 4. Conclusions

To summarise, thermosensitive, injectable smart hydrogel LDDSs were developed and thoroughly investigated. The hydrogels were produced from PCEC triblock copolymers, which were successfully synthesized via ROP with CL and PEG in the presence of Zr(acac)_4_ as a catalyst. It was discovered that the Zr(acac)_4_ catalyst exhibits outstanding activity in the polymerization of CL and, due to its improved biosafety profile, may be an interesting alternative to tin-based catalysts, which are typically used in these types of reactions.

The method of PCEC triblock copolymers synthesis with used of Zr-catalyst was repeatable. Given the low M_n_ values, it is reasonable to conclude that the obtained copolymers have an acceptable *Đ* index. The optimal polymerization conditions for ROP of CL in the presence of PEG were determined to be 130 °C and 6 h; the CL/Zr(acac)_4_ molar ratio was 1000:1. The high percentage of monomer conversion and adequate yield values demonstrated that Zr(acac)_4_ catalyst is sufficient in the ROP process.

In the case of thermosensitive hydrogel formulation, the most favourable PCEC copolymer properties were observed for macromolecules containing PEG 1000 as a hydrophilic block with a CL/PEG ratio close to 2.0 [m/m]. For these copolymers, the sharp sol–gel transition was clearly observed at the temperature slightly lower than physiological, ensuring a rapid (<5 min) formation of stable gel with good mechanical properties (G′ ≈ 90,000 Pa for 20 wt% PCEC-A2.0 hydrogel) at 37 °C. The optimal PCEC concentration was determined to be 20 wt% to 30 wt%. These findings appear to be consistent with previous research on PCEC injectable hydrogels [3,17].

The analysis of the 5-FU release profiles reveals that the release follows first order kinetics, with a release time of 12 h. Fitting the data to the Higuchi and Korsmeyer–Peppas mathematical models indicates that the diffusion mechanisms of the antitumor drug are clearly predominant. The hydrophilic nature of the 5-FU and the matrix clearly explains this phenomenon.

Finally, injectable smart hydrogels capable of releasing a hydrophilic antitumor drug were developed. The obtained hydrogels appear promising as short term LDDSs, potentially useful in antitumor therapy, due to the repeatable and well-controlled release rate, as well as the biodegradability and biosafety of PCEC.

## Figures and Tables

**Table 1:** PCEC triblock copolymers synthesis and characterization.

				Feed Ratio			NMR					GPC ^e^				
Sample	Time [h]	PEG M_n_ [g/mol]	CL/PEG Ratio [m/m]	M_PCL_^a^	M_PEG_^a^	M_n_^a^	M_PCL_^a^	M_PEG_^a^	n_CL_^b^	n_EG_^b^	M_n_^a^	M_n_^a^	*Đ* ^c^	Yield [%]	LCST ^d^ [°C]	UCST ^d^ [°C]
PCEC-1	6	200	1.9	380	200	580	1000	200	8.6	4.2	1200	1700	1.53	59	not soluble	
PCEC-2	6	200	1.5	300	200	500	800	200	7.2	4.0	1000	1900	1.53	65	not soluble	
PCEC-3	6	200	1.1	220	200	420	800	200	7.0	4.3	1000	2000	1.60	65	not soluble	
PCEC-4	6	600	2.0	1200	600	1800	2000	600	17.2	12.9	2500	3100	1.45	87	not soluble	
PCEC-5	6	600	1.7	1020	600	1620	2000	600	17.0	13.4	2500	3000	1.57	95	not soluble	
PCEC-6	6	600	1.3	780	600	1380	1600	700	14.2	14.9	2300	2800	1.54	91	not soluble	
PCEC-7	6	1000	2.0	2000	1000	3000	2900	1000	25.8	22.0	3900	4200	1.55	85	**38**	**51**
PCEC-8	12	1000	2.0	2000	1000	3000	2800	1000	24.2	22.3	3700	4300	1.53	99	43	52
PCEC-9	6	1000	2.0	2000	1000	3000	3100	1100	27.0	24.1	4100	4800	1.56	94	**45**	**51**
PCEC-10	24	1000	2.0	2000	1000	3000	2400	900	21.4	21.0	3400	3700	1.51	92	36	51
PCEC-11	6	1000	1.7	1700	1000	2700	2400	1000	21.4	23.5	3500	3500	1.54	83	37	50
PCEC-12	6	1000	1.3	1300	1000	2300	2100	1000	18.4	23.0	3100	3400	1.53	79	37	49
PCEC-13	6	1500	2.7	4050	1500	5550	6700	1700	59.0	38.6	8400	7400	1.53	90	not soluble	
PCEC-14	6	1500	2.0	3000	1500	4500	4000	1700	35.4	37.7	5700	6200	1.48	96	soluble	
PCEC-15	6	1500	1.3	1950	1500	3450	3200	1700	27.8	37.9	4800	5300	1.45	87	soluble	
PCEC-16	6	2000	2.7	5400	2000	7400	7100	2000	62.4	46.9	9200	7900	1.44	63	not soluble	
PCEC-17	6	2000	2.0	4000	2000	6000	5400	2200	47.2	50.1	7600	6900	1.52	94	not soluble	
PCEC-18	6	2000	1.3	2600	2000	4600	4000	2300	34.8	52.9	6300	6600	1.53	99	not soluble	
PCEC-A1.7	6	1000	1.7	1700	1000	2700	2000	1000	17.2	23.5	3000	3300	2.05	93	29	47
PCEC-A2.0	6	1000	2.0	2000	1000	3000	2500	1000	21.4	22.5	3400	3500	2.26	95	33	48

^a^ Number average molecular weight [g/mol] of PCL (M_PCL_), PEG (M_PEG_) or copolymer (M_n_). ^b^ The average number of CL or ethylene glycol mers. ^c^ Dispersity. ^d^ Lower critical solution temperature (LCST) and upper critical solution temperature (UCST). The gelation behavior was evaluated for 20 wt% (or 30 wt% for PCEC-7 and PCEC-9) triblock copolymer aqueous solution by tube-inverting method. ^e^ Gel permeation chromatography (GPC).

**Table 2:** Optimization of the CL/catalyst molar ratio.

			Feed Ratio		^1^H NMR		Percent Agreement with Theoretical Values [%]	
Sample	Monomer/Catalyst Molar Ratio [mol/mol]	CL/PEG Ratio [m/m]	M_PCL_ [g/mol]	M_n_ [g/mol]	M_PCL_ [g/mol]	M_n_ [g/mol]	M_n_	M_n_ of PCL Block
PCEC-12	100	1.3	1300	2300	2100	3100	134	159
CAT-1	1125	1.3	1300	2300	1000	1900	84	77
CAT-2	1250	1.3	1300	2300	700	1900	84	74
PCEC-11	100	1.7	1700	2700	2400	3500	129	144
CAT-3	250	1.7	1700	2700	2200	3300	121	132
CAT-4	500	1.7	1700	2700	2100	3100	116	125
CAT-5	750	1.7	1700	2700	2000	2900	108	115
CAT-6	1000	1.7	1700	2700	1900	2800	104	112
CAT-7	1125	1.7	1700	2700	1500	2400	91	87
CAT-8	1250	1.7	1700	2700	1800	2700	102	107
PCEC-7	100	2.0	2000	3000	2900	3900	130	145
CAT-9	250	2.0	2000	3000	2600	3600	119	128
CAT-10	500	2.0	2000	3000	2500	3500	116	124
CAT-11	750	2.0	2000	3000	2000	2800	92	101
CAT-12	1000	2.0	2000	3000	2200	3100	103	109
CAT-13	1125	2.0	2000	3000	2100	3000	100	103
CAT-14	1250	2.0	2000	3000	1700	2600	88	84

**Table 3:** The ROP kinetics over a time period of 0.5–6 h.

Sample	Time [h]	M_PCL_^a^ [g/mol]	M_PEG_^a^ [g/mol]	M_PCL_^a^ [g/mol]	M_n_^a^ [g/mol]	CL Conv.^a^ [%]	Yield [%]	M_n_^b^ [g/mol]	*Đ* ^b^
PCEC-T0.5	0.5	500	1000	500	2100	89	35	2300	1.28
PCEC-T1	1	300	1200	300	1700	88	23	2100	1.28
PCEC-T2	2	500	1000	500	2100	90	61	3000	1.24
PCEC-T3	3	700	1000	700	2300	91	55	3300	1.30
PCEC-T4	4	1200	1000	1200	3400	95	70	2800	2.47
PCEC-T5	5	1000	1000	1000	3000	94	82	2400	2.45
PCEC-T6	6	1200	1000	1200	3300	99	88	3300	2.19

^a^ Calculated from ^1^H NMR spectra. ^b^ Determined from GPC.

**Table 4:** Data analysis of 5-FU release from hydrogels.

Sample	Zero Order	First Order	Higuchi		Korsmeyer–Peppas	
	R^2^	R^2^	R^2^	K_H_^b^	R^2^	*n* ^c^
A	0.783	**0.911 ^a^**	0.915	19.082	0.999	0.397
B	0.791	**0.906 ^a^**	0.920	20.241	0.998	0.500
C	0.755	**0.842 ^a^**	0.878	13.374	0.924	0.332

^a^ R^2^ values indicating zero order or first order kinetics of the 5-FU release from the hydrogel were bolded. ^b^ Release rate constant (Higuchi model). ^c^ The release exponent (Korsmeyer-Peppas model).

## Data Availability

The data presented in this study are available on request from the corresponding author.

## Supplementary Materials

The following are available online at https://www.mdpi.com/article/10.3390/ijms22158330/s1.
