# Supplementary material for: Synthesis and Characterization of New Biodegradable Injectable Thermosensitive Smart Hydrogels for 5-Fluorouracil Delivery
Source: Int J Mol Sci. 2021 Aug 3;22(15):8330. doi: 10.3390/ijms22158330 (PMC8347305; doi:10.3390/ijms22158330)
Supplement: Supplementary file 1 [file ijms-22-08330-s001.zip › ijms-1286804-supplementary.pdf]

International Journal of
Molecular Sciences

MDPI

Supplementary Materials

Table S1. Cytotoxicity assay of the PCEC copolymers (for the highest concentrations tested (1 mg/mL)).

Sample	Cells survival ± SD [%]
PCEC-A1.7	96 ± 1
PCEC-A2.0	98 ± 4
Positive control	0 ± 0
Negative control	103 ± 7

Table S2. Genotoxicity assay of PCEC-A1.7 and PCEC-A2.0 samples (performed at the highest concentration (1 mg/mL), with and without metabolic activation).

	-S9[1]		+S9[2]	
Sample	G ± SD	IR ± SD	G ± SD	IR ± SD
PCEC-A1.7	1.03 ± 0.16	0.92 ± 0.08	1.07 ± 0.17	0.86 ± 0.17
PCEC-A2.0	0.98 ± 0.18	0.98 ± 0.18	1.05 ± 0.09	0.91 ± 0.20
Solvent control	0.95 ± 0.06	1.00 ± 0.01	1.02 ± 0.05	0.95 ± 0.00
Positive control	0.94 ± 0.00	2.31 ± 0.10	0.93 ± 0.05	1.59 ± 0.01
Negative control	1.00 ± 0.03	1.00 ± 0.07	1.00 ± 0.05	1.00 ± 0.08

[1] without metabolic activation, [2] with metabolic activation

Table S3. HPLC gradient for 5-fluorouracil analysis.

Time [min]	Phase A [%] (H_2O + 0.1 % TFA)	Phase B [%] (ACN + 0.1 % TFA)
0	99	1
7	96	4
12	20	80
22	20	80
27	99	1
33	99	1
